# Large Bowel Obstruction in a Young Woman Simulating a Malignant Neoplasm: A Case Report of *Actinomyces* Infection

**DOI:** 10.1155/2013/756768

**Published:** 2013-06-27

**Authors:** R. Nissi, R. B. Blanco Sequeiros, E. Lappi-Blanco, H. Karjula, A. Talvensaari-Mattila

**Affiliations:** ^1^Department of Obstetrics and Gynecology, Oulu University Hospital, University of Oulu, P.O. Box 24, 90029 Oulu, Finland; ^2^Department of Radiology, Oulu University Hospital, 90029 Oulu, Finland; ^3^Department of Pathology, Oulu University Hospital, 90029 Oulu, Finland; ^4^Department of Surgery, Oulu University Hospital, 90029 Oulu, Finland

## Abstract

Pelvic and intra-abdominal Actinomycosis can be difficult to diagnose preoperatively and it may also mimic many other diseases, including malignancies. We present a patient with pelvic Actinomycosis probably caused by a long-standing intrauterine device (IUD). We emphasize the challenges in diagnostic process and stress that though a rare disease, intra-abdominal Actinomycosis should be suspected in cases with intra-abdominal mass of uncertain etiology. The early recognition may spare the patient from extensive surgical operation.

## 1. Introduction

Pelvic Actinomycosis is a rare disease with poorly understood pathogenesis. It commonly occurs in women with an intrauterine device of prolonged duration. *Actinomyces* are Gram-positive anaerobic bacteria that are normal inhabitants of the mouth and bowel. The organism only penetrates mucosal barrier after a tissue injury [1].

Early diagnosis of abdominal Actinomycosis is of importance, as timely recognition enables conservative treatment with antibiotics, avoiding surgery [2]. There is no documented person-to-person transmission of the disease [3].

## 2. Case Report

Our own case is a 45-year-old woman, who was referred to surgical ward due to an intestinal occlusion and tumor-like mass in the region of flexura lienalis. The patient had no preceding chronic disease or constant medication. She complained of increasing lower abdominal discomfort for 6 months with diarrhea and constipation. 

**Figure 1:**
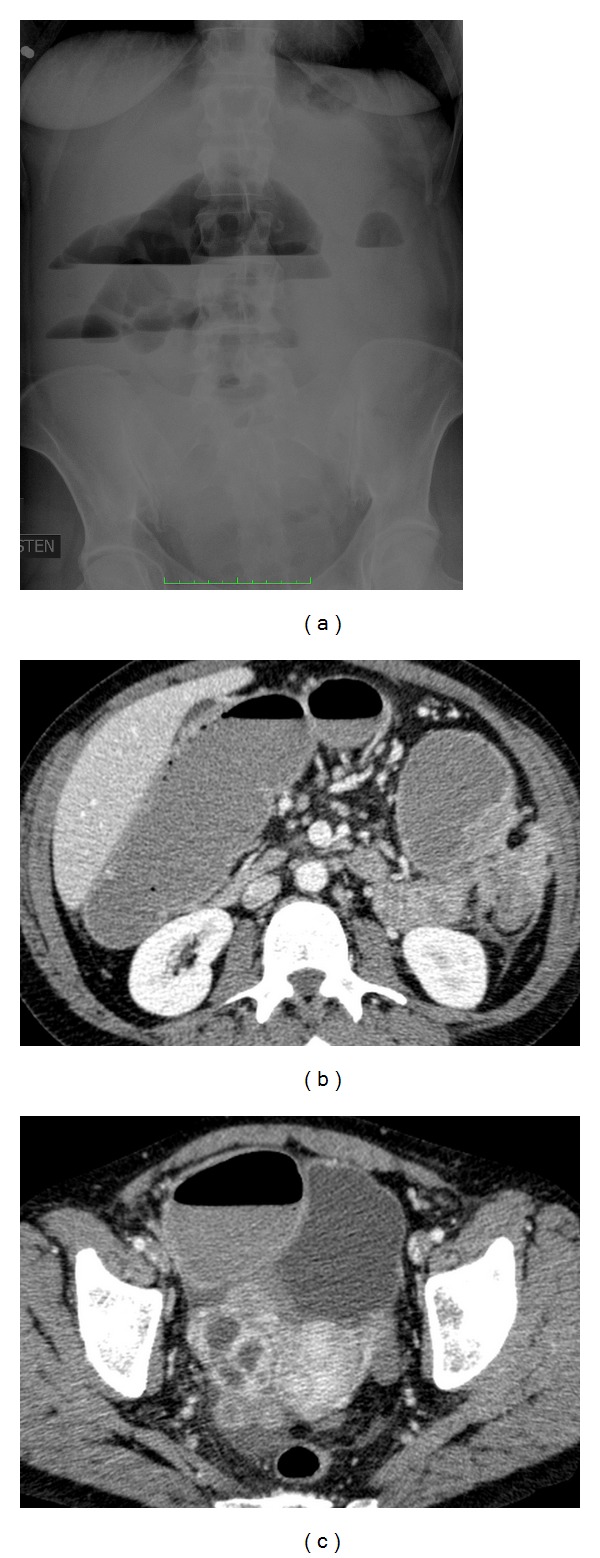
(a) An abdominal radiograph reveals multiple fluid levels and a dilated bowel segment (arrow heads), (b) a CT scan from upper abdomen exhibits dilated colon structure (black arrowheads), narrowed colonic segment (white arrowhead), and extraluminal infiltration (arrow), and (c) A CT scan from lower abdomen exhibits cystic involvement in right ovary (arrowheads) as well as enlarged colon (asterisk).

**Figure 2:**
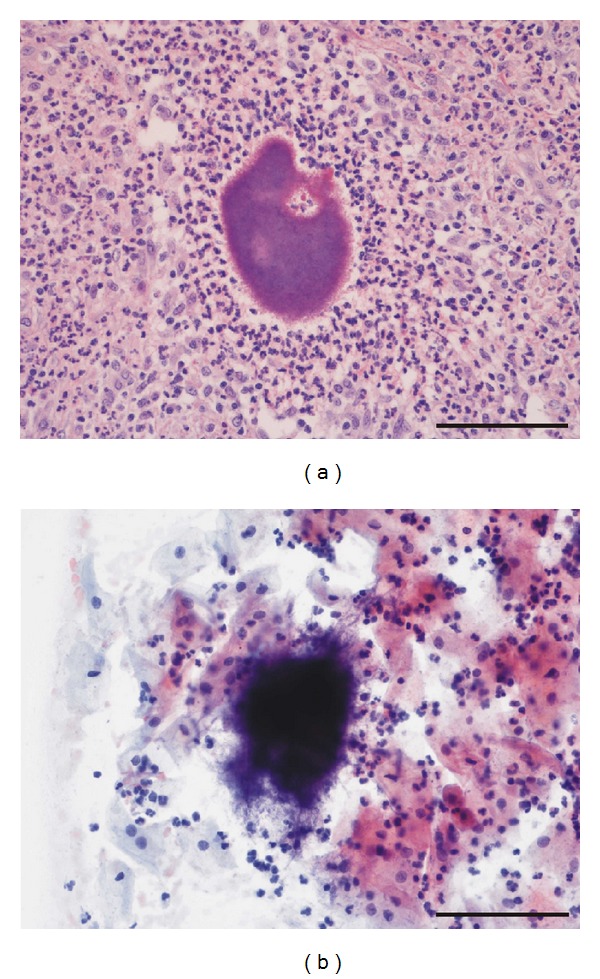
Cotton ball-like bacterial aggregates consistent with *Actinomyces* species infection in an HE-stained section of an ovarian abscess (a) and a Papanicolaou-stained cervical smear (b). Bar = 0.1 mm.

Abdominal native radiographs suggested occlusion (Figure 1). Laboratory parameters showed an increased thrombocyte count (689 × 10^9^/L) and a mild decrease of hemoglobin value (107 g/L). Other laboratory parameters including C-reactive protein, leukocytes, CEA, CA 12-5, and CA 19-9 were normal. She had no weight loss or fever. A contrast-enhanced (i.v.) 16-detector row helical pelvic CT scan was performed (Figure 2). Bowel contrast was not used. CT showed dilated colon (maximal diameter 8 cm) and, distal to this, a narrowed colon segment at flexura lienalis, presenting as a probable cause of occlusion. There was tissue infiltration surrounding the bowel stricture as well as separate omental infiltration proximal and distal to the obstruction site. Peritoneal fat stranding, indicating edema, was present surrounding the abnormal bowel segments and there was an enhancing cystic lesion (diameter 4.5 cm) in the right ovary. There were also increased number of lymph nodes in paraaortil space and omentum. Malignancy could not be equivocally excluded. Because of a tumor-like mass and prolonged intensive occlusion lasting for 10 days, a laparotomy was performed. There was a 20 cm tumour-like mass in flexura lienalis infiltrating omentum and causing occlusion. In lower abdomen a tumor of 7 cm diameter infiltrating subcutaneous tissue was observed. Left ovary was inflamed without signs of malignancy. There was no ascites. Expanded hemicolectomy and jejunal resection were performed. A gynecologist performed a right-side salpingo-oophorectomy. 

Macroscopic gross pathology examination of the formalin-fixed tissues revealed inflamed right ovary and tuba, focal damage of colonic mucosa, and wide peritoneal fibrosis and adhesions with pus. Histology showed purulent salpingo-oophoritis, necrosis of colonic mucosa, chronic peritoneal inflammation with abscess formation, granulation tissue, and diffuse fibrosis. Multiple Gram-positive cotton ball-like bacterial aggregates consistent with *Actinomyces* species infection were observed (Figure 2(a)). Postoperative endometrial biopsy and vaginal and cervical smears also revealed aggregates of *Actinomyces* species (Figure 2(b)). The patient has had one pregnancy with successful delivery 21 years ago. At that time an IUD was inserted without gynecological followup. The microbiological analysis of removed IUD revealed bacterial growth of *E. Coli *and* S. epidermidis*. The patient was treated with intravenous penicillin (20 megaunits/day) and peroral metronidazole (1.5 g/day) for 7 weeks. After that, peroral amoxicillin (1.5 g/day) was conducted for 6 months. Papanicolaou test revealed ASC-US, but after 6 months the results were normal.

## 3. Discussion

The most difficult task in the management of abdominal Actinomycosis is to reach diagnosis before surgical operation takes place. Actinomycosis has been shown to mimic appendiceal tumor and abdominopelvic actinomycosis is associated with abdominal surgery and bowel perforation or occlusion [4]. If diagnosed preoperatively, antibiotic treatment may lead to complete recovery. Exclusive antibiotic treatment for pelvic Actinomycosis has also been reported [3], but if occlusion is present, an operative treatment is justified. Also the possibility of malignancy favors surgical treatment.

We report a case of pelvic Actinomycosisin a patient using the same IUD for over 20 years. The Actinomycosiscaused occlusion and appeared as a pelvic mass simulating malignancy and was surgically removed. Subsequently, the patient was treated with appropriate antibiotics with good result. Penicillin is the antibiotic of choice for treatment of Actinomycosis infection, whereas metronidazole appears to be most effective in treating associated infection of Gram-negative organisms. A prolonged treatment is required because of the poor penetration of antibiotics into the inflamed and fibrotic tissue [3].

According to the literature, the typical patient at risk for IUD-associated *Actinomyces* infection is 37 years old with IUD usage lasting longer than 8 years. A long-standing IUD can also cause perforation of the uterus [5]. The clinical symptoms are characterized by chronic intermittent or subacute abdominal pain, weight loss, vaginal discharge, and fever. 

Laboratory parameters commonly revealed anemia and leukocytosis. Imaging findings are nonspecific. Typically, a neoplastic or inflammatory process is suggested. CT is the most useful imaging study as it reveals the extent of the disease. Most common CT features at abdominopelvic [6] region include bowel wall thickening and solid mass with possible cystic elements. Despite advanced imaging and diagnostics, most patients undergo operative procedure. 

Even though intra-abdominal Actinomycosis is very rare, it should be included in a list of differential diagnoses, especially in any women using IUDs with abdominal pain and pelvic mass. This may spare the patient from unnecessary surgery.
